# The malaria testing and treatment landscape in mainland Tanzania, 2016

**DOI:** 10.1186/s12936-017-1819-7

**Published:** 2017-04-24

**Authors:** ACTwatch Group, Louis Akulayi, Angela Alum, Andrew Andrada, Julie Archer, Ekundayo D. Arogundade, Erick Auko, Abdul R. Badru, Katie Bates, Paul Bouanchaud, Meghan Bruce, Katia Bruxvoort, Peter Buyungo, Angela Camilleri, Emily D. Carter, Steven Chapman, Nikki Charman, Desmond Chavasse, Robyn Cyr, Kevin Duff, Gylsain Guedegbe, Keith Esch, Illah Evance, Anna Fulton, Hellen Gataaka, Tarryn Haslam, Emily Harris, Christine Hong, Catharine Hurley, Whitney Isenhower, Enid Kaabunga, Baraka D. Kaaya, Esther Kabui, Beth Kangwana, Lason Kapata, Henry Kaula, Gloria Kigo, Irene Kyomuhangi, Aliza Lailari, Sandra LeFevre, Megan Littrell, Greta Martin, Daniel Michael, Erik Monroe, Godefroid Mpanya, Felton Mpasela, Felix Mulama, Anne Musuva, Julius Ngigi, Edward Ngoma, Marjorie Norman, Bernard Nyauchi, Kathryn A. O’Connell, Carolyne Ochieng, Edna Ogada, Linda Ongwenyi, Ricki Orford, Saysana Phanalasy, Stephen Poyer, Justin Rahariniaina, Jacky Raharinjatovo, Lanto Razafindralambo, Solofo Razakamiadana, Christina Riley, John Rodgers, Andria Rusk, Tanya Shewchuk, Simon Sensalire, Julianna Smith, Phok Sochea, Tsione Solomon, Raymond Sudoi, Martine Esther Tassiba, Katherine Thanel, Rachel Thompson, Mitsuru Toda, Chinazo Ujuju, Marie-Alix Valensi, Vamsi Vasireddy, Cynthia B. Whitman, Cyprien Zinsou, Daniel Michael, Sigsbert Patila Mkunde

**Affiliations:** 1 Population Services International, 1120 19th St NW Suite 600, Washington, DC 20036 USA; 2 PSI/Tanzania, Plot # 1347/48 Masaki, Msasani Peninsula, Haile Selassie Road, PO Box 33500, Dar es Salaam, Tanzania; 3 National Malaria Control Programme, Dar es Salaam, United Republic of Tanzania

**Keywords:** Quality-assured artemisinin combination therapy, ACT, Rapid diagnostic testing, Anti-malarial markets

## Abstract

**Background:**

Understanding the key characteristics of malaria testing and treatment is essential to the control of a disease that continues to pose a major risk of morbidity and mortality in mainland Tanzania, with evidence of a resurgence of the disease in recent years. The introduction of artemisinin combination therapy (ACT) as the first-line treatment for malaria, alongside policies to promote rational case management following testing, highlights the need for evidence of anti-malarial and testing markets in the country. The results of the most recent mainland Tanzania ACTwatch outlet survey are presented here, including data on the availability, market share and price of anti-malarials and malaria diagnosis in 2016.

**Methods:**

A nationally-representative malaria outlet survey was conducted between 18th May and 2nd July, 2016. A census of public and private outlets with potential to distribute malaria testing and/or treatment was conducted among a representative sample of administrative units. An audit was completed for all anti-malarials, malaria rapid (RDT) diagnostic tests and microscopy.

**Results:**

A total of 5867 outlets were included in the nationally representative survey, across both public and private sectors. In the public sector, availability of malaria testing was 92.3% and quality-assured (QA) ACT was 89.1% among all screened outlets. Sulfadoxine–pyrimethamine (SP) was stocked by 51.8% of the public sector and injectable artesunate was found in 71.4% of all screened public health facilities. Among anti-malarial private-sector stockists, availability of testing was 15.7, and 65.1% had QA ACT available. The public sector accounted for 83.4% of the total market share for malaria diagnostics. The private sector accounted for 63.9% of the total anti-malarial market, and anti-malarials were most commonly distributed through accredited drug dispensing outlets (ADDOs) (39.0%), *duka la dawa baridi* (DLDBs) (13.3%) and pharmacies (6.7%). QA ACT comprised 33.1% of the national market share (12.2% public sector and 20.9% private sector). SP accounted for 53.3% of the total market for anti-malarials across both private and public sectors (31.3 and 22.0% of the total market, respectively). The median price per adult equivalent treatment dose (AETD) of QA ACT in the private sector was $1.40, almost 1.5 times more expensive than the median price per AETD of SP ($1.05). In the private sector, 79.3% of providers perceived ACT to be the most effective treatment for uncomplicated malaria for adults and 88.4% perceived this for children.

**Conclusions:**

While public sector preparedness for appropriate malaria testing and case management is showing encouraging signs, QA ACT availability and market share in the private sector continues to be sub-optimal for most outlet types. Furthermore, it is concerning that SP continues to predominate in the anti-malarial market. The reasons for this remain unclear, but are likely to be in part related to price, availability and provider knowledge or preferences. Continued efforts to implement government policy around malaria diagnosis and case management should be encouraged.

**Electronic supplementary material:**

The online version of this article (doi:10.1186/s12936-017-1819-7) contains supplementary material, which is available to authorized users.

## Background

Following declines in malaria prevalence in the first decade of the twenty first century, more recent data from mainland Tanzania have shown evidence of a resurgence in the disease. Among children under five, malaria prevalence halved from 18 to 9% between 2007–2008 and 2011–2012, but has since risen to 14%. There is also regional variation, with prevalence as high as 28% in the Western zone [1]. Ninety-three percent of mainland Tanzania’s population resides in malaria-endemic areas, and in 2015 there were estimated to be 7.3 million clinical and confirmed cases of malaria reported in the country [2].

Tanzania Mainland’s Strategic Plan for Malaria 2015–2020, includes goals to (1) reduce malaria illness and deaths by 80.0% from 2012 levels; (2) reduce malaria prevalence to 1.0%; and, (3) increase the proportion of pregnant women receiving two or more doses of sulfadoxine–pyrimethamine (SP) during pregnancy to 80.0% [3]. Malaria case management priorities are: to improve the quality of diagnostic and case management services; to maintain and improve anti-malarial drug supplies in the public sector; to improve access to quality and affordable artemisinin combination therapy (ACT) in the private sector. The strategy further outlines specific areas of focus that will support these targets, including strengthening the supply chain, information provision and behaviour change communications (BCC) to promote universal diagnostic coverage and uptake of ACT.

The 2014 Tanzania Mainland’s National Guidelines for the Diagnosis and Treatment of Malaria stipulate artemether–lumefantrine (AL) as the first-line treatment for uncomplicated malaria in both adults and children, with dihydroartimisinin-piperaquine (DHA PPQ) as a second-line treatment in cases of treatment failure [4]. The guidelines were also updated to align with the World Health Organization (WHO) recommendations stipulating injectable (IV/IM) artesunate for treatment of patients with severe malaria, and a three-course treatment of SP for intermittent treatment as prevention during pregnancy (IPTp) (rather than a two-course treatment as previously recommended). According to the 2014 National Guidelines, patients with severe malaria should be referred to a public health facility. Quinine is the second-line treatment for cases of uncomplicated malaria contra-indicated for ACT and for women in the first trimester of pregnancy, or in cases of severe malaria not responding to first-line treatment. The 2014 National Guidelines also advocate parasitological confirmation of suspected malaria cases for all ages in mainland Tanzania. Since 2006, oral artemisinin monotherapy has been banned [5].

Mainland Tanzania has implemented several strategies in recent years to improve access to confirmatory testing and first-line ACT treatment. For example, between 2007 and 2013, the mainland Tanzanian public sector received 93.1 million doses of AL [3]. Between 2009 and 2012, a phased roll-out of malaria rapid diagnostic tests (RDTs) to all levels of government health facilities was implemented to complement microscopy services, with national coverage in 2013 [6]. This was in line with growing recognition that relatively inexpensive and sensitive RDTs could be made available at the most peripheral levels of the public health sector.

In 2010, mainland Tanzania participated in the Affordable Medicines Facility-malaria (AMFm) pilot, administrated by the Global Fund, with the aim of increasing access to ACT and reducing use of artemisinin monotherapy in the public and private sector [7]. ACT that achieved accredited status from the WHO, European Medicines Authority (EMA) or the Global Fund (termed quality assured [QA] ACT) were subsidized at ‘factory gate’ before entering the supply chains in countries involved in the project. AMFm-subsidized products carried a ‘green leaf’ logo to differentiate them from non-subsidized and non-QA ACT products [8]. Following the AMFm pilot period in 2010–2011, the subsidy mechanism transitioned into a new model called the private sector co-payment mechanism (CPM) which continued to fund ACT subsidies in the private sectors of many malaria-endemic countries, including mainland Tanzania [8, 9]. The CPM focused exclusively on the private sector supply of QA ACT given that an independent evaluation showed that the AMFm had greater impact on the supply of QA ACT in the private than the public sector [10]. The public sector continued to receive subsidized ACT through an alternative Global Fund mechanism, and QA ACT medicines in this sector were not marked with the ‘green leaf’ logo.

In the 12 month period prior to data collection reported here, 7.3 million treatment doses were delivered in mainland Tanzania during the CPM period, representing a decline from the AMFm peak—where 21.6 million doses were delivered in 2012 (personal communication, Global Fund). The CPM period was further marked by a reduction in the level of subsidy to first-line ACT buyers—from over 90% during the AMFm period to 70%~ in 2016. During the CPM period in mainland Tanzania, there was an absence of any provider or consumer behaviour change communication or other supporting interventions to increase awareness of the subsidized, QA ACT (personal communication, Global Fund).

Several other initiatives have focused on improving malaria case management services in the private sector. Following the loosening of laws in mainland Tanzania in 1991 that had previously banned the provision of medical services in the private sector, there was a proliferation and diversification of private providers [11]. A large proportion of outlets were registered (but essentially unregulated) private medicine dispensing outlets, including small drug shops called *duka la dawa baridi* (DLDBs) (sometimes referred to as Part II drug outlets) [12]. According to the national policy, DLDBs are only permitted to sell non-prescription medications [13]. However, in practice they frequently dispensed prescription-only treatments. In 2003, the mainland Tanzanian government introduced the accredited drug dispensing outlet (ADDO) programme, which aimed to provide accreditation to these outlets, through a programme of training and support to increase their capacity to provide quality primary health services, particularly in remote areas [14]. As part of the accreditation process, ADDO providers received training on malaria case management and malaria national treatment guidelines [13]. ADDOs are permitted to sell prescription medicines, including ACT, while referring any cases of severe malaria to a public health facility. Since a successful 2012 pilot initiative introducing testing with RDTs in ADDOs, efforts have been made to begin a national roll-out of RDTs in these outlets. There are over 4000 ADDOs located mainly in rural areas [15], and thought to be another 2000 outlets awaiting accreditation by the government nationally.

Understanding the anti-malarial and malaria diagnostic supply side will be an important means to inform future case management strategies and guide programmes aimed at improving adherence to national guidelines. Since 2010, ACTwatch has been implementing outlet surveys in mainland Tanzania to generate timely, relevant and high quality evidence about anti-malarial markets for policy makers, donors and implementing organizations [16]. In 2016, ACTwatch implemented its final survey in mainland Tanzania. The objective of this paper is to provide practical evidence to inform strategies and policies in mainland Tanzania towards achieving national malaria control goals. The paper describes the total market for malaria medicines and diagnostics at national level.

## Methods

### Design and sampling

The 2016 mainland Tanzania outlet survey was a nationally representative, cross-sectional, quantitative survey conducted among a sample of outlets stocking anti-malarial medicines and diagnostics. The survey was implemented between 18 May and 2 July, 2016. This was the fourth such survey conducted in mainland Tanzania.

Detailed ACTwatch project and methodological information have been published elsewhere [16, 17]. Briefly, all potential outlet types stocking anti-malarials and diagnostics in mainland Tanzania in both public and private sectors were included in the study. According to the ACTwatch methodology, outlets are included in the survey if they have the ‘potential’ to sell or distribute anti-malarials or diagnostic testing. This includes outlets that may not typically be expected to stock anti-malarial medicines, such as general retailers, village shops, or itinerant drug vendors. However, it is recognized that in many countries these outlets can operate as vendors for anti-malarial commodities, either illegally or/and outside of the formal health system. These outlets are included in the sample as a means to confirm their role or presence in a given country’s anti-malarial and diagnostic market. These outlets may differ on a country-by-country basis, but broad categories are used to define public and private sector outlets.

Outlets sampled in the public sector included public health facilities (e.g., the national referral hospital, regional hospitals, district hospitals, health centres, dispensaries), and private not-for-profit facilities [including non-governmental organizations (NGOs) hospitals and clinics, faith-based hospitals, clinics]. The private-sector outlet types sampled were private for-profit health facilities (including private hospitals, clinics, diagnostic laboratories), pharmacies (which are registered and licensed by a national regulatory authority, and staffed by pharmacists and qualified health practitioners), ADDOs (drug stores that primarily sell medicines, registered with a national regulatory authority, where staff have received training), DLDBs (drug stores that primarily sell medicines, with no formal licensing, and no guarantee of staff training), general retailers (grocery stores and village shops), and itinerant drug vendors (mobile, unregistered providers selling medicines).

The primary sampling approach taken for ACTwatch outlet surveys entails sampling a set of administrative units (geographic clusters) with a population of approximately 10,000 to 15,000 inhabitants. Clusters are selected with cluster probability of selection proportionate to size (PPS). The most appropriate administrative unit in mainland Tanzania matching the desired population size was at the ward level.

Clusters (wards) were selected using probability proportional to population size sampling, using data from the 2012 Tanzania Population and Housing Census [18]. Additional wards were selected for oversampling of public health facilities, private for-profit health facilities, pharmacies, and ADDOs. This booster sampling strategy was used to obtain a sufficient sample size for indicator estimates within these outlet types. The sample was stratified by urban–rural ward designation. In total, 58 wards were selected for the main census sample (28 rural, 30 urban) and a further 172 wards were selected for the booster sample (84 rural, 88 urban).

Within each selected cluster a census of all outlet types with the potential to provide anti-malarials or diagnostics to consumers was undertaken. The inclusion criteria for outlets were: (1) one or more anti-malarials in stock on the day of the survey; (2) one or more anti-malarials in stock in the three months preceding the survey; and, (3) malaria blood testing (RDTs or microscopy) available.

### Sample size

The study was powered to detect a minimum of a 20% point change in availability of QA ACT among anti-malarial stockists between each round and within each domain for each survey, at the 5% significance level with 80% power. The number of study clusters was calculated for each research domain based on the required number of anti-malarial stockists and assumptions about the number of anti-malarial stockists per cluster. Sample size requirements for the 2016 survey were calculated using information from the 2014 survey round including anti-malarial and QA ACT availability, outlet density per cluster, and design effect.

### Training and fieldwork

Fieldworker training consisted of standardized classroom presentations and exercises as well as a field exercise. Examinations administered during training were used to select field workers, supervisors and quality controllers. Additional training was provided for supervisors and quality controllers focused on field monitoring, verification visits and census procedures. Fieldwork teams were provided with a list of selected clusters and official maps that illustrated administrative boundaries. In each selected cluster, fieldworkers conducted a full enumeration of all the aforementioned outlet types. This included enumeration of outlets with a physical location, as well as identification of itinerant drug vendors using local informants. To identify outlets, fieldworkers systematically walked through each cluster, looking for the outlets. To distinguish between pharmacies, ADDOs and DLDBs, fieldworkers were trained to look for licenses hanging up on the wall and to prompt providers for any clarification, especially when these licenses were not legible. In mainland Tanzania, pharmacies have licenses clearly displayed above counters, and ADDOs have a specific license that include a logo to recognise the outlet as part of the programme. The primary provider/owner of each outlet was invited to participate in the study and the screening questions were administered to assess anti-malarial and diagnostic availability.

Interviews were conducted in Swahili using questionnaires that were translated from English to Swahili and back to English to confirm translations. A structured questionnaire programmed into mobile phones using DroidDB software was used to complete an audit of all anti-malarials and RDTs as well as a provider interview. Quality control measures implemented during the fieldwork included questionnaire review by supervisors. Up to 20% of all outlets were also checked by quality controllers to verify the interview had been completed.

### Protection of human subjects

The 2016 outlet survey protocol received ethical approval from the national ethical approval board in mainland Tanzania (Reference number: NIMR/HQ/R.8a/Vol. IX/2209). Provider interviews and product audits were completed only after administration of a standard informed consent form and provider consent to participate in the study. Providers had the option to end the interview at any point during the study. Standard measures were employed to maintain provider confidentiality and anonymity.

### Measures

The outlet survey questionnaire included an audit of all available anti-malarial medicines and RDTs. Providers were asked to show the interviewer all anti-malarial medicines and RDTs currently available. A product audit sheet captured information for each unique product in the outlet, including formulation, brand name, active ingredients and strengths, package size, manufacturer and country of manufacture for anti-malarials, and brand name, manufacturer, country of manufacture, antigens and parasite species for RDTs. Providers were asked to report the retail and wholesale price for each product as well as the amount distributed to individual consumers in the last week.

### Data analysis and indicators

Data were analysed using Stata (StataCorp College Station, TX, USA). Standard indicators were constructed according to definitions applied across the ACTwatch project, descriptions of which have been provided in detail elsewhere [9, 11]. Anti-malarials identified during the outlet drug audit were classified according to information on drug formulation, active ingredients and strengths as non-artemisinin therapy, artemisinin monotherapy and ACT. Non-artemisinin therapy was classified as SP or other non-artemisinin therapy. Although no longer indicated for malaria case management, SP is still recommended for IPTp. Artemisinin monotherapy was further classified as oral and non-oral, the latter including medicines recommended for first-line treatment of severe malaria. ACT was classified as QA ACT or non-quality-assured ACT. QA ACT were ACT granted World Health Organization (WHO) prequalification, ACT in compliance with the Global Fund Quality Assurance Policy, on the Global Fund list of approved pharmaceutical products for procurement, or ACT granted regulatory approval by the European Medicines Agency (EMA). Classification was completed by matching product audit information (formulation, active ingredients, strengths, manufacturer, country of manufacture, package size) to lists of approved medicines from the WHO, EMA and Global Fund.

QA ACT availability in the public sector was among all outlets screened, while in the private sector it was restricted to those outlets that had anti-malarials in stock. Anti-malarial market share, or the relative distribution of the anti-malarials to individual consumers recorded in the drug audit, was standardized to allow for meaningful comparisons between anti-malarials with different treatment courses and different formulations. The adult equivalent treatment dose (AETD) was defined as the amount of active ingredient required to treat an adult weighing 60 kg according to WHO treatment guidelines [19]. Provider reports on the amount of the drug sold or distributed during the week preceding the survey were used to calculate volumes according to type of anti-malarial. The volume of each drug was calculated as the number of AETDs that were reported to have been sold/distributed during the week preceding the survey. Measures of volume included all dosage forms to provide a complete assessment of anti-malarial market share. Diagnostic market share was calculated from the number of malaria blood tests (i.e., microscopy and RDT) performed or distributed by outlet type as a proportion of all tests performed or distributed in the previous week.

Median private sector price for one AETD was calculated for QA ACT and for the most popular non-artemisinin therapy, which in mainland Tanzania was SP given it was the most common non-artemisinin therapy distributed. The interquartile range (IQR) is displayed as a measure of dispersion. Price data presented were collected in local currencies and converted to US dollar prices (average exchange rate for the data collection period). Price measures included tablet anti-malarials only, given differences in unit costs for tablet and non-tablet formulations. While all QA ACT are by definition tablet formulations, SP may be available in other formulations including syrups and injections. These other formulations were excluded from median price calculations.

Provider knowledge was measured as the percentage of providers who identified ACT as the most effective treatment for uncomplicated malaria. This was measured separately for adults and children, and is reported here by outlet type; 95% confidence intervals provide an indicator of the precision of the estimates.

Sampling weights were calculated as the inverse of the probability of cluster selection. All point estimates were weighted using survey settings and all standard errors calculated taking account of the clustered and stratified sampling strategy with the relevant suite of survey commands in Stata. A finite population correction was also applied to adjust standard errors, as a relatively large proportion of available clusters were selected for inclusion in the sample.

## Results

### Sample description

A total of 5868 outlets were screened for availability of anti-malarials and/or malaria blood testing services and 2,317 were subsequently interviewed. A total of 2194 outlets surveyed were found to have anti-malarials in stock on the day of the survey, 39 had anti-malarials reportedly in stock during the previous 3 months but not on the day of the visit, and 84 had malaria blood testing available but no antimalarials in stock (A more detailed breakdown of outlet sample eligibility, by rural/urban strata may be found in Additional file 1).

### Availability

Table 1 shows the availability of anti-malarials and malaria diagnosis among all screened public sector outlets. Across the public sector, 96.2% had any anti-malarial on the day of the survey. QA ACT availability was 92.0% among public health facilities and 65.8% among private not-for-profit facilities. When disaggregated by pack size (Additional file 2), availability of paediatric QA AL was 62.2% in public health facilities and 23.2% in private not-for-profit facilities. The availability of non-QA ACT was 13.3% in public health facilities and 29.8% in private not-for profit outlets. SP accounted for the majority of available non-artemisinin therapy, stocked by 51.8% of the public sector. Injectable artesunate was found in 71.4% of public health facilities and 24.9% private not-for-profit facilities.

**Table 1:** Availability of anti-malarials and malaria testing among all screened public sector outlets

	Public health facility % CI	Private not for-profit facility % CI	Public sector total % CI
	N = 341	N = 65	N = 406
Any anti-malarial	96.2 (89.0, 98.8)	96.4 (79.5, 99.5)	96.2 (90.0, 98.6)
QA ACT	92.0 (84.0, 96.2)	65.8 (49.0, 79.4)	89.1 (82.5, 93.5)
non-QA ACT	13.3 (7.5, 22.5)	29.8 (17.1, 46.7)	15.1 (9.3, 23.6)
SP	51.5 (40.3, 62.6)	54.1 (36.9, 70.4)	51.8 (41.5, 61.9)
Oral Quinine	4.6 (1.8, 11.3)	51.4 (33.9, 68.6)	9.7 (6.3, 14.6)
Other non-artemisinin therapy (amodiaquine and parenteral quinine)	9.7 (6.5, 14.2)	9.3 (3.7, 21.4)	9.7 (6.7, 13.9)
Artesunate injection	71.4 (62.9, 78.5)	24.9 (16.0, 36.6)	66.3 (57.8, 73.8)
Any malaria testing	91.8 (85.6, 95.4)	97.1 (80.6, 99.6)	92.3 (86.7, 95.7)
Malaria microscopy	18.9 (13.3, 26.2)	68.9 (54.1, 80.6)	24.3 (18.2, 31.8)
RDTs	89.3 (82.2, 93.7)	89.3 (77.4, 95.3)	89.3 (82.9, 93.4)
QA ACT and any malaria testing	86.0 (77.9, 91.5)	62.9 (46.4, 76.8)	83.5 (76.4, 88.8)
QA ACT no malaria testing	5.9 (2.8, 12.1)	2.9 (0.4, 19.4)	5.6 (2.7, 11.2)

Malaria diagnostics were available in 91.8% of public health facilities (18.9% had microscopy and 89.3% stocked RDTs) and in 97.1% of private not for-profit facilities (68.9% had microscopy and 89.3% had RDTs).

Across the public sector, availability of both QA ACT and testing was 83.5%, and this was higher in public health facilities (86.0%) than private not-for-profit facilities (62.9%).

In the private sector, among all screened outlets, availability of any anti-malarial was highest among pharmacies (99.2%; N = 61), ADDO (96.9%; N = 1503) and DLDB (94.7%, N = 148). Of the 3541 general retailers screened, only 0.5% had anti-malarials in stock (Additional file 3).Table 2 shows private sector availability of different types of anti-malarials among outlets with any anti-malarial in stock on the day of the survey. Among anti-malarial stockists, 65.1% of the private sector had QA ACT available. In terms of availability of different pack sizes, 52.7% had adult pack sizes of QA AL in stock and 20.4% had paediatric QA AL (Additional file 4). Non-QA ACT was found in 42.9% of all anti-malarial stocking private outlets. ACT was most commonly available in anti-malarial stocking pharmacies (QA ACT 90.0%; non-QA ACT 98.7%). SP was the most commonly available non-artemisinin therapy anti-malarial in the therapy stocking private sector, and over 90% of pharmacies and ADDOs had SP in stock. Oral quinine was also stocked by 64.6% of private sector outlets. Injectable artesunate was available in 34.4% of private for-profit health facilities and 17.9% of pharmacies, but was otherwise largely absent from the private sector (Table 2).

**Table 2:** Availability of anti-malarials and malaria testing among anti-malarial stocking private sector outlets

	Private for-profit facility % CI	Pharmacy % CI	ADDO % CI	DLDB % CI	Private sector total % CI
	N=118	N=60	N=1468	N=142	N=1800
QA ACT	73.6 (63.0, 82.0)	90.0 (75.0, 96.4)	66.3 (57.7, 74.0)	58.8 (42.8, 73.1)	65.1 (57.2, 72.3)
Non-QA ACT	66.5 (49.0, 80.4)	98.7 (94.2, 99.7)	43.3 (33.1, 54.1)	34.9 (21.3, 51.4)	42.9 (31.5, 55.1)
SP	76.8 (70.0, 82.4)	94.4 (90.2, 96.9)	92.2 (87.9, 95.0)	75.2 (62.2, 84.9)	86.3 (80.4, 90.6)
Oral quinine	63.9 (49.0, 76.6)	74.8 (61.7, 84.5)	65.2 (57.7, 72.1)	67.8 (51.5, 80.6)	64.6 (57.9, 70.8)
Other non-artemisinin therapy	8.1 (3.9, 16.2)	0.7 (0.1, 3.4)	2.6 (1.3, 5.3)	6.1 (2.6, 14.0)	4.0 (2.3, 7.0)
IV/IM artesunate	34.4 (26.9, 42.9)	17.9 (5.6, 44.5)	0.2 (0.1, 0.8)	0.0 (–)	2.4 (1.4, 4.0)

	N=120	N=61	N=1490	N=146	N=1832
Any test	96.0 (91.1, 98.3)	21.9 (12.8, 34.8)	10.2 (6.4, 15.9)	8.1 (3.9, 16.2)	15.7 (12.6, 19.4)
Microscopy	73.9 (51.0, 88.5)	8.8 (1.8, 34.3)	0.6 (0.2, 2.3)	0.0 (–)	5.4 (2.8, 10.3)
RDT	78.5 (69.4, 85.4)	21.9 (12.8, 34.8)	9.7 (6.0, 15.3)	8.1 (3.9, 16.2)	14.3 (11.4, 17.7)

**Table 3:** Median private sector price (and IQRs) for anti-malarials and malaria blood testing

	N	Median price (USD)	IQR
QA ACT	2251	$1.40	[1.24–1.86]
Non-QA ACT	1381	$4.65	[1.5–6.25]
SP	4239	$1.05	[0.93–1.40]
Microscopy (adult)	88	$0.70	[0.47–0.93]
Microscopy (child)	88	$0.47	[0.47–0.93]
RDT (adult)	247	$0.93	[0.47–0.93]
RDT (child)	247	$0.93	[0.47–0.93]

Malaria diagnosis was available among 15.7% of the anti-malarial stocking private sector, and highest among private for-profit facilities (96.0%), followed by pharmacies (21.9%), ADDOs (10.2%) and DLDBs (8.1%). Availability of malarial microscopy was 5.4%; availability of RDTs was 14.3% in the private sector.

### Price

The median price per AETD of QA ACT in the private sector was $1.40, and almost 1.5 times more expensive than the median price per AETD of SP ($1.05) (Table 3). The median price per AETD of non-QA ACT was $4.65. When disaggregated by outlet type, the price of these three anti-malarials was usually lower in DLDBs than other private outlet types (Additional file 5).

The median prices for malaria microscopy in the private sector was $0.70 for an adult and $0.47 for children. The median malaria RDT price for an adult and child was $0.93.

### Anti-malarial market share

**Fig. 1:**
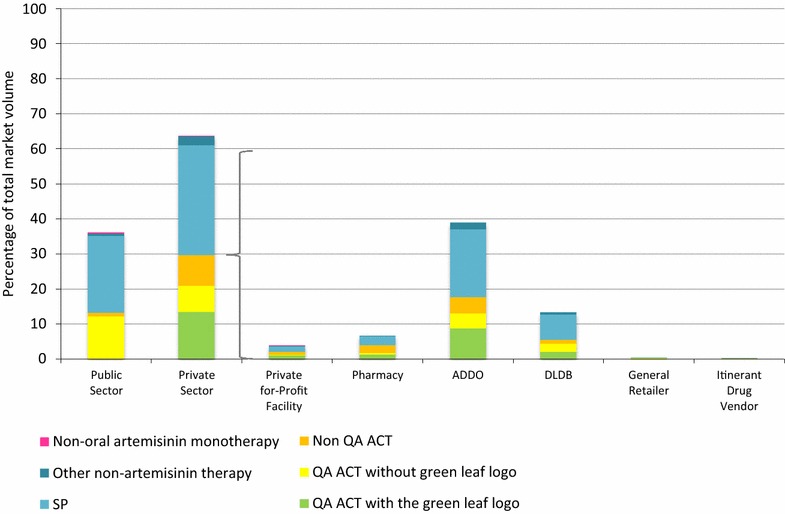
Anti-malarial market share

The public sector accounted for 36.1% of all anti-malarial volumes distributed in the week prior to the survey. Of all anti-malarials distributed, 12.2% were QA ACT in the public sector, with public sector SP accounting for a further 22.0% (Fig. 1).

The private sector accounted for 63.9% of the total anti-malarial market, and anti-malarials were most commonly distributed through ADDOs (39.0%), DLDBs (13.3%) and pharmacies (6.7%). Of all the anti-malarials distributed, 29.7% were ACT in the private sector (QA ACT with the logo, 13.5%; 7.4 QA ACT without the logo and 8.8% non-QA ACT). Most of the private sector ACT was distributed through ADDOs (17.7%). SP was the most commonly distributed anti-malarial in the private sector (31.3%). Oral AMT was absent from the market in this survey round.

The relative anti-malarial market share within outlet type is shown in Additional file 6. Of note is the similarity in anti-malarial mix between ADDOs and DLDBs, with ACT making up 45.4 and 41.8% of their distributed anti-malarials, respectively.

### Diagnostic market share

**Fig. 2:**
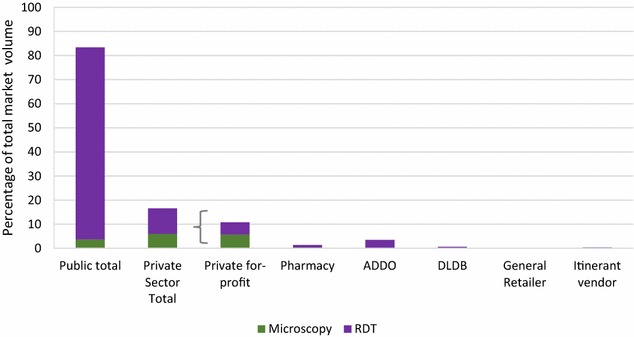
Diagnostic market share

The public sector accounted for 83.4% of the total market share for malaria diagnostics (Fig. 2). Most of the diagnostic tests distributed across the public and private sector were RDTs (90.2% of the total diagnostic market share).

### Provider perceptions

**Fig. 3:**
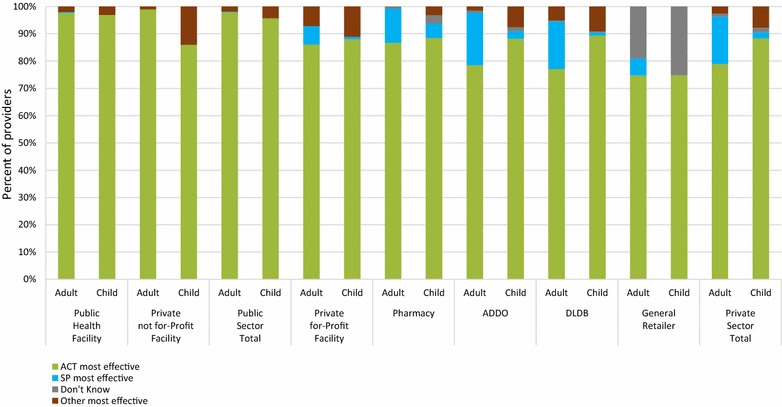
Provider perceptions of the most effective treatment for uncomplicated malaria

Figure 3 shows the percentage of providers who reported that ACT was the most effective treatment for uncomplicated malaria for adults and children. Providers in the public sector perceived ACT as the most effective treatment for uncomplicated malaria for adults and children (97.9 and 95.7%, respectively). In the private sector, 79.3% of providers perceived ACT to be the most effective treatment for uncomplicated malaria for adults and 88.4% perceived this for children. Almost one in five providers working in ADDOs and DLDBs perceived that ACT was not the most effective treatment for adults (21.1 and 22.9%, respectively).

## Discussion

This paper has provided a comprehensive overview of the malaria testing and treatment landscape in mainland Tanzania in 2016, in both public and private sectors. While the public sector shows strong readiness to adhere to national guidelines, there is sub-optimal QA ACT availability and market share in the private sector. There is also persistent widespread distribution of SP, which continues to predominate the anti-malarial market.

### Public sector readiness for malaria case management

The results indicate that in terms of availability, the public sector’s level of readiness for appropriate malaria case management is high. The National Malaria Strategic Plan [3] aims for the provision of universal access to malaria testing and first-line treatment, and these results indicate that universal access has almost been achieved in this sector. Almost every screened outlet in the public sector had QA ACT available and over 90% of public sector outlets had either malaria microscopy or RDTs available, reflecting several strategies implemented nationally since 2013 to scale up confirmatory testing in this sector. Only a small fraction (5.6%) of outlets had QA ACT available without testing, and this signifies an improvement since 2014 (where 9.5% of public sector outlets had QA ACT but no testing) [20]. Furthermore, three-quarters of public health facilities had injectable artesunate, the first-line treatment for severe malaria, and this reflects a substantial increase in the public sector since the previous survey round, from 21.3% in 2014 [20] to 66.3% in 2016. National efforts to align with the WHO recommendations for treatment of severe malaria are reflected in these findings. The results for provider knowledge in the public sector were also encouraging, and stand in contrast to a previous study that found overall poor levels of knowledge of AL in this sector [21].

Despite these promising findings, there are some gaps in public sector readiness for malaria case management that require attention. Of concern is the finding that SP was available in just over half of all screened outlets in the public sector, meaning that much of the public sector is not equipped to provide IPTp, although this reflects an increase from 2011 and 2014 [22]. This is also in light of several national strategies that have encouraged universal coverage of IPTp during pregnancy and substantial roll-out of IPTp3+ nationwide. The findings from the most recent outlet survey suggest there are key challenges to be addressed, including maintaining a constant supply of SP across the public health sector. This will be important to address in order to meet national targets that stipulate 80% coverage of IPTp by 2020.

In addition, despite widespread availability of QA ACT and sub-optimal availability of SP, the market share findings illustrate that SP was more widely administered than QA ACT in the public sector. QA ACT market share within the public sector was also at its lowest level since before the launch of the AMFm, only one in three anti-malarials distributed in the public sector were a QA ACT in 2016 compared to one in every two in 2010 [22]. These findings may reflect stock-outs of different pack sizes of QA ACT. While the strength of all first-line AL tablets for treatment of uncomplicated malaria is indeed the same, the implementation of the AL policy includes delivery of four different AL pack sizes (6, 12, 18 and 24 tablets) suitable for management of four different weight categories of patients (5–14; 15–24; 25–34; ≥35 kg). In the public sector, availability of the different weight categories was variable. For example, a pack size of 12 tablets of QA AL was available in less than half of the public sector outlets. Providers may ration ACTs because of uncertainty with supply coupled with availability of non-recommended treatments [23]. Alternatively, this may reflect an increase of RDTs in this sector and better management of patients through confirmatory testing, lending to a reduction of QA ACT market share. Other population based evidence from mainland Tanzania between 2010 and 2012 reported a significant decrease in the percentage of people with fever obtaining ACT from 57.4 to 46.1%, along with an increase in the percentage of people obtaining a blood test from 28.7 to 46.6% [24]. As such, the market share findings from this outlet survey may reflect increases in diagnostic coverage and better management of patients.

### The role of the private sector in malaria case management

In 2016, almost all pharmacies, ADDOs and DLDBs that were screened were in the business of stocking malaria commodities, as were around three-quarters of private not for-profit facilities. Consistent with previous outlet surveys [20], general retailers are not typically anti-malarial stockists. Of the 3540 screened outlets, only ten had anti-malarials in stock in 2016. Market share data also illustrate the importance of the private sector, which accounted for 63.9% of the total anti-malarial market. Anti-malarials in the private sector were also most commonly distributed through ADDOs.

The concentration of malaria commodities distributed among ADDOs may reflect several strategies to license DLDBs. Since 2003, the ADDO programme has been implemented as a means to regulate and improve service provision of health care in the private sector. The findings from 2016 illustrate that ADDOs accounted for over 1468 anti-malarials stockists in the private sector compared to 142 DLDBs, representing the greatest concentration of private sector service delivery points for malaria. This reflects a change in market composition from previous surveys, where in 2010 most of the private sector anti-malarial service delivery points were DLDBs (48% of the total market composition) as compared to ADDOs (20% of the market composition) [22]. These findings are reflective of the several initiatives by the mainland Tanzania government over the years to scale up the ADDO programme and increase coverage of regulated private sector outlets. As of 2015, between 4000 and 9000 DLDBs had been accredited, becoming ADDOs, nationally in mainland Tanzania [15].

### Readiness and performance of the private sector for malaria case management

Where anti-malarials were available in the private sector, just over half of the anti-malarial stockists had QA ACT available. Market share data also illustrated that in 2016 around 30% of the private sector market share comprised ACT, reflecting an overall increase from 2010 [22]. Non-artemisinin therapy, typically SP, accounted for one-half of all anti-malarials distributed. The availability of malaria confirmatory testing was also very low in the private sector. This is corroborated by household survey data that found only 2.1% of febrile children under five received a confirmatory test in the private sector [24]. However, where confirmatory testing was available, the results also demonstrate that the median price of all malaria diagnostics was lower than QA ACT, which is encouraging as it may provide a cost incentive for a patient to test before treatment.

While ADDOs accounted for the largest distribution of QA ACT, and comprised most of the market share in the private sector, there was very little difference in the anti-malarial mix across outlet types, given SP was the most commonly dispensed anti-malarial across all outlets. Lessons on how to maintain and improve ACT availability and distribution among these outlet types can be learnt from several studies that have investigated factors which influence ADDO ACT stocking characteristics. Studies have found that ADDOs with greater client load and which are in close proximity to other outlets that sell ACT medicines, are more likely to stock ACT as compared to isolated outlets which serve fewer customers [25, 26]. Another determinant of ACT stocking practices among ADDOs has been the presence of a licensed pharmacist [25, 26], and somewhat related to this, staff retention. One study found that up to 40% of trained ADDO dispensers were no longer working at the outlet, and consequently other untrained staff were employed lending to poor dispensing practices, irrational use of medicines, and even poorer performance than DLDBs [27]. Future strategies to improve the retention rate of trained personnel at ADDOs will be key to ensure the sustainability of an effective ADDO programme and may want to consider the targeting of busier outlets in competitive markets to encourage faster uptake of ACT.

In addition to their role in the provision of anti-malarial medication, ADDOs are now permitted to perform testing (using RDTs) for malaria. Nevertheless, there is little to differentiate ADDOs from their unregulated counterparts in terms of malarial blood testing availability, with RDT stocking levels languishing below 10%. Indeed, this mirrors challenges documented in other countries, where maintaining constant supply of RDTs have been noted, as well as determining effective incentives for private providers and patients to use RDTs and adhere to results [28]. Despite these challenges, several studies have documented the feasibility of including RDT testing in ADDOs. For example, a randomized controlled trial to investigate whether the introduction of RDTs among ADDOs improved malaria case management found that confirmatory diagnosis increased from 19 to 74% in intervention districts, which also resulted in improved targeting of ACT to patients with malaria [29]. Similar positive outcomes have been demonstrated in other countries, [30, 31], with studies concluding that private sector outlets can safely and correctly test for malaria with appropriate training, supervision, and record keeping [32]. Scaling up access to RDTs in the private sector through ADDOs will be an important initiative to improve levels of confirmatory testing and treatment practices. Of promise is that the mainland Tanzania National Strategic Plan includes strategies to improve accessibility and affordability of RDTs by facilitating the procurement of quality diagnostic devices at subsidized/low costs through the global partnership [3]. The findings from the 2016 study provide a benchmark from which this can be measured.

Given evidence from this survey that ADDOs are the most important private sector outlet in the provision of anti-malarials, future strategies can target these outlets as a means to disrupt the widespread distribution of SP for case management, increase uptake of ACT and RDTs. Future training and learning opportunities provide an opportunity to emphasize the importance of adhering to national treatment guidelines, address misconceptions that SP is the most effective treatment for adults, and ensure constant supply of QA ACT and RDTs to these providers. In short, there is significant opportunity with regard to the role that ADDOs play in the rationalization of malaria treatment and diagnosis, particularly given their predominance in the anti-malarial private sector market and current efforts to engage with this sector.

### Market performance of the green leaf ACT logo

The results from the most recent survey illustrate that the market share of ACT remained less than 50% and ACT carrying the ‘green leaf’ logo (a marker of subsidized QA ACT) was less than 15%. This also reflected a decline from 2014 levels, which was the result of an upward trend in market share since the introduction of the subsidy mechanism [10]. These findings are perhaps not surprising considering the transition from a dedicated donor funding during the AMFm period to a country specific grant funding mechanism, which was further amplified by a reduction in funding for co-paid ACT. Indeed, the number of subsidized QA ACT doses delivered to mainland Tanzania’s private sector through the CPM was a third of what it was in 2012. Furthermore, the ACT price subsidy in 2016 was 70% compared to ~90% during the AMFm period, lending to an increase in QA ACT price over the years [22] such that the price of QA ACT was one and a half times that of SP in 2016. In this context, the reduction in the green leaf logo market share to the relatively low levels reported in this paper may largely be explained by a more limited supply and availability of these medications in the context of reduced funding and scaled down programming. Furthermore, in the absence of supportive interventions, including behaviour change communications designed to increase awareness of the QA ACT with the logo, providers and consumers alike may have less awareness of the first-line, subsidized treatment. Indeed, the results from this study illustrated that in the private sector ACT is still not universally perceived as the most effect treatment for uncomplicated malaria. Up to one in five providers continued to cite treatments that were not ACTs. In absence of supportive interventions targeted at both consumers and providers to raise awareness of affordable, quality, first-line treatment for malaria, behaviour change will be challenging [33].

### Availability and distribution of non-QA ACT

In the private sector, availability and distribution of non-QA ACT was common. One in every three ACT medicines distributed were non-QA ACT, and distribution was most common among private for-profit facilities and pharmacies as compared to other private sector outlet types. This is of concern given that quality-assurance status has been associated with high quality medicines in drug quality studies [34]. In Tanzania, a nationally representative survey of over 1700 anti-malarials in the private sector found that ACT samples lacking WHO prequalification were 25 times more likely to be of poor quality than those with WHO prequalification status [35], illustrating how quality-assurance status can serve as an important indicator of ACT drug quality.

While public sector outlets may be required to obtain particular drugs that meet certain quality standards, quality may not necessarily be a factor in private sector procurement decisions. This may in part explain why the private sector was found to stock and distribute non-QA ACT. What is of interest however is that non-QA ACT was three times more expensive that QA ACT, yet it was still being distributed, indicating that some consumers were willing to pay over four USD for a treatment. This raises the question of why consumers would pay more for non-QA ACT when less expensive QA ACT are available. Non-QA ACT products were primarily available and distributed by private for-profit facilities and pharmacies, which are more common anti-malarial service delivery points in urban areas as compared to rural areas in Tanzania [22]. As outlets located in urban areas typically serve consumers with a higher socio-economic status [22, 36], these consumers may be able to better afford the relatively high price of non-QAACT. Or, this could be related to a lack of awareness of subsidy programme, given demand creation strategies had not been promoted in the past several years. Indeed, a better understanding of provider and consumer demand for QA ACT and non-QAACT will be important for developing strategies to promote use of QA ACT over non-QA products.

### Availability and use of SP

The results from the 2015 illustrate the widespread availability and distribution of SP, and this is a barrier to implementation of the government policy for first-line treatments for uncomplicated malaria. Furthermore, while SP is mandated for use in IPTp, it seems likely from these results that it is also being utilized more widely than government policy recommends. Government efforts to encourage universal coverage of IPTp during pregnancy may have driven increased demand for SP, and there is some evidence that levels of IPTp have increased in recent years [1]. However, while there has been a substantial roll-out of IPTp3+ nationwide, with parallel behaviour change communication and promotion through public health facilities, there is no evidence or policy documents suggesting that the private sector should also play a role in the provision of this service in the country. Continued uses of SP likely include management of fever/malaria in people of all ages given the widespread availability and distribution of this anti-malarial.

The substantial private sector SP market share is cause for concern, and suggests it is being administered for malaria case management, against national (and international) guidelines. This is also supported by other evidence that suggests many SP products have packaging and patient instructions indicating its use for uncomplicated malaria for all ages. Additional file 7 shows some example photographs of SP packaging collected during fieldwork, some of which clearly indicate that the product is appropriate for the treatment of malaria for all ages. Other research in mainland Tanzania has corroborated these findings and illustrated that SP was predominantly distributed to men [37].

### Limitations

The results presented in this study provided a cross-sectional snapshot of the anti-malarial testing and treatment markets in mainland Tanzania in 2016. The ACTwatch outlet survey design has limitations that have been documented and reported elsewhere [17, 38]. Limitations specific to the mainland Tanzania study centre around potential bias emerging from interviews with DLDBs, for whom the practice of supplying prescription medications is not permitted. All vendors participating in this study gave their informed consent to take part, and were assured of their anonymity. It is feasible that vendors may deflate or increase the levels of anti-malarial testing or treatment that they are reporting. The use of an electronic data collection approach, while convenient from a data collection perspective, may have had the effect of arousing suspicion among the interviewees. In addition, the study was not designed or powered to compare ADDOs and DLDBs, but it did provide an opportunity to examine these outlet types and explore their performance. In addition, it is uncertain that these two outlet types were substantively different, as DLDBs may have begun, but not yet completed the accreditation process at the time of the survey, or indeed may have competed the process previously and then lost their accreditation.

## Conclusion

Tanzania is in a unique position in that several strategies have been in place to improve malaria case management services and this paper provides a contemporary understanding of mainland Tanzania’s anti-malarial landscape. Overall, mainland Tanzania’s public sector is well prepared for malaria testing and treatment, with good levels of provider knowledge. The private sector however appears to have several gaps in its preparedness, which is reflective of reduced funding levels for the subsidy programme since the AMFm. QA ACT availability and market share in the private sector continues to be disappointing for most outlet types, and there is persistent widespread distribution of SP, which continues to predominate in the market. The reasons for this remain unclear, but are likely to be related to overall reduced funding of the ACT subsidy programme, such that affordable and more widely available SP remains in favourable competition to QA ACT. In the absence of supportive interventions, provider and consumer knowledge of the first-line treatment are also a barrier. Government drives for increased IPTp, while encouraging, are unlikely to fully explain the high levels of SP distribution. Continued efforts to implement government policy around malaria diagnosis and case management should be encouraged.

## Additional files

**Additional file 1.** Detailed breakdown of the sample.

**Additional file 2.** QA AL availability in the public sector, by pack size.

**Additional file 3.** Availability of anti-malarials among all private sector screened outlets.

**Additional file 4.** QA AL Availability in the private sector, by pack size.

**Additional file 5.** Price for all private sector outlets.

**Additional file 6.** Antimalarial market share, within outlet type.

**Additional file 7.** SP product photographs from fieldwork.

## Abbreviations

ADDO: accredited drug dispensing outlet
AETD: adult equivalent treatment dose
ACT: artemisinin-based combination therapy
AMFm: affordable medicines facility for malaria
AL: artemether lumefantrine
BCC: behaviour change communications
CPM: co-payment mechanism
DHA-PP: dihydroartemisinin piperaquine
DLDB: *duka la dawa baridi*
EMA: European Medicines Authority
IPTp: intermittent treatment as prevention during pregnancy
IQR: interquartile range
NGO: non-government organization
SP: sulfadoxine pyrimethamine
PPS: probability proportionate to size
RDT: rapid diagnostic test
QA: quality assured
WHO: World Health Organization

## References

[1] ICF Marco, MoHSW. Tanzania Demographic and Health Survey and Malaria Indicator Survey (TDHS–MIS) 2015–2016. Dar es Salaam: MoHSW; 2016. https://www.dhsprogram.com/pubs/pdf/FR321/FR321.pdf. Accessed 24 Feb 2017.

[2] PMI. Tanzania 2016 President’s Malaria initiative fact sheet; 2016. https://www.usaid.gov/documents/1860/tanzania-2016-presidents-malaria-initiative. Accessed 24 Feb 2017.

[3] NCMP. The United Republic of Tanzania National Malaria Strategic Plan 2014–2020; 2014.

[4] MoHSW (2014). National guidelines for diagnosis and treatment of malaria.

[5] WHO. World malaria report. Geneva: WHO; 2016. http://apps.who.int/iris/bitstream/10665/252038/1/9789241511711-eng.pdf?ua=1. Accessed 24 Feb 2017.

[6] CHAI. Case study: increasing availability of malaria rapid diagnostic tests in Tanzania’s private sector; 2015. http://www.clintonhealthaccess.org/content/uploads/2015/10/Malaria_RDTs_AXP_Case_Study_vFINAL_101515.pdf. Accessed 24 Feb 2017.

[7] Independent Evluation Team. Independent evaluation of phase 1 of the affordable medicines facility-malaria (AMFm), multi-country independent evaluation report: final report. Calverton: ICF International and London School of Hygiene and Tropical Medicine; 2012. https://www.theglobalfund.org/en/technical-evaluation-reference-group/evaluations/evaluation-2013-2014/. Accessed 4 Apr 2017.

[8] Global Fund. Private sector co-payment mechanism; 2016. http://www.theglobalfund.org/en/sourcing/privatesectorcopayment/. Accessed 24 Feb 2017.

[9] Global Fund. Integration of the Lessons from the Affordable Medicines Facility-malaria. https://www.theglobalfund.org/board-decisions/b28-dp06/. Accessed 7 Apr 2017.

[10] Tougher S, ACTwatch Group, Ye Y, Amuasi JH, Kourgueni IA, Thomson R, Goodman C (2012). Effect of the affordable medicines facility-malaria (AMFm) on the availability, price, and market share of quality-assured artemisinin-based combination therapies in seven countries: a before-and-after analysis of outlet survey data. Lancet.

[11] SHOPS Project. Tanzania Private Health Sector Assessment. Bethesda: Brief; 2013. http://shopsproject.org/sites/default/files/resources/Tanzania%20Private%20Sector%20Assessment%202.pdf. Accessed 24 Feb 2017.

[12] Ndomondo-Sigonda RM, Shirima R, Heltzer N, Clark M, Ndomondo-Sigonda R. Improving access to quality drugs and services in rural and periurban areas with few or no pharmacies; 2004. http://archives.who.int/icium/icium2004/resources/ppt/AC105.pdf. Accessed 24 Feb 2017.

[13] Management Sciences for Health. Accredited drug dispensing outlets in Tanzania: strategies for enhancing access to medicines program; 2008. http://www.drugsellerinitiatives.org/publication/altview/addo-in-tanzania-strategies-for-enhancing-access-to-medicines-program-final-report/PDF/. Accessed 24 Feb 2017.

[14] MoHSW (2003). National Health Policy.

[15] PMI. Tanzania malaria operational plan 2016; 2016. https://www.pmi.gov/docs/default-source/default-document-library/malaria-operational-plans/fy16/fy-2016-tanzania-malaria-operational-plan.pdf?sfvrsn=4. Accessed 24 Feb 2017.

[16] Shewchuk T, O’Connell KA, Goodman C, Hanson K, Chapman S, Chavasse D (2011). The ACTwatch project: methods to describe anti-malarial markets in seven countries. Malar J..

[17] O’Connell KA, Gatakaa H, Poyer S, Njogu J, Evance I, Munroe E (2011). Got ACTs? Availability, price, market share and provider knowledge of anti-malarial medicines in public and private sector outlets in six malaria-endemic countries. Malar J.

[18] MoH (2015). Tanzania population and housing census 2015.

[19] WHO. Guidelines for the treatment of malaria. Third edition. Geneva: WHO; 2015. http://www.who.int/malaria/publications/atoz/9789241549127/en/.

[20] ACTwatch Group. ACTwatch Study Reference Document Tanzania Outlet Survey; 2014. http://www.actwatch.info/sites/default/files/content/publications/attachments/Tanzania%20Outlet%20Report%202014.pdf. Accessed 24 Feb 2017.

[21] Kamuhabwa AA, Silumbe R (2013). Knowledge among drug dispensers and antimalarial drug prescribing practices in public health facilities in Dar es Salaam. Drug Healthc Patient Saf..

[22] ACTwatch Group. ACTwatch Study Reference Document Tanzania Outlet Survey; 2016.

[23] Rao VB, Schellenberg D, Ghani AC (2013). Overcoming health systems barriers to successful malaria treatment. Trends Parasitol..

[24] Thomson R, Festo C, Johanes B, Kalolella A, Bruxvoort K, Nchimbi H (2014). Has Tanzania embraced the green leaf? Results from outlet and household surveys before and after implementation of the Affordable medicines facility-malaria. PLoS ONE.

[25] O’Meara WP, Obala A, Thirumurthy H, Khwa-Otsyula B (2013). The association between price, competition, and demand factors on private sector anti-malarial stocking and sales in western Kenya: considerations for the AMFm subsidy. Malar J.

[26] Larson PS, Yadav P, Alphs S, Arkedis J, Massaga J, Sabot O (2013). Diffusion of subsidized ACTs in accredited drug shops in Tanzania: determinants of stocking and characteristics of early and late adopters. BMC Health Serv Res.

[27] Minzi O, Manyilizu V (2013). Application of basic pharmacology and dispensing practice of antibiotics in accredited drug-dispensing outlets in Tanzania. Drug Healthc Patient Saf.

[28] Yeung S, Patouillard E, Allen H, Socheat D (2011). Socially-marketed rapid diagnostic tests and ACT in the private sector: ten years of experience in Cambodia. Malar J.

[29] Maloney K, Ward A, Krenz B, Petty N, Bryson L, Dolkart C (2017). Expanding access to parasite-based malaria diagnosis through retail drug shops in Tanzania: evidence from a randomized trial and implications for treatment. Malar J.

[30] Ansah EK, Narh-Bana S, Affran-Bonful H, Bart-Plange C, Cundill B, Gyapong M (2015). The impact of providing rapid diagnostic malaria tests on fever management in the private retail sector in Ghana: a cluster randomized trial. BMJ (Clin Res Ed)..

[31] Mbonye AK, Clarke SE, Lal S, Chandler CI, Hutchinson E, Hansen KS (2015). Introducing rapid diagnostic tests for malaria into registered drug shops in Uganda: lessons learned and policy implications. Malar J..

[32] Ansah EK, Narh-Bana S, Epokor M, Akanpigbiam S, Quartey AA, Gyapong J (2010). Rapid testing for malaria in settings where microscopy is available and peripheral clinics where only presumptive treatment is available: a randomised controlled trial in Ghana. BMJ (Clin Res Ed)..

[33] Willey BA, Tougher S, Ye Y, Mann AG, Thomson R, Kourgueni IA (2014). Communicating the AMFm message: exploring the effect of communication and training interventions on private for-profit provider awareness and knowledge related to a multi-country anti-malarial subsidy intervention. Malar J..

[34] Bate R, Hess K (2012). The role of pre-shipment batch testing in ensuring good medicine quality. Malar World J..

[35] IMPACT2 Study Team (2015). Quality of artemisinin-containing antimalarials in Tanzania’s Private Sector–results from a Nationally Representative Outlet Survey. Am J Trop Med Hyg.

[36] van der Hoeven M, Kruger A, Greeff M (2012). Differences in health care seeking behaviour between rural and urban communities in South Africa. Int J Equity Health.

[37] Mikkelsen-Lopez I, Tediosi F, Abdallah G, Njozi M, Amuri B, Khatib R (2013). Beyond antimalarial stock-outs: implications of health provider compliance on out-of-pocket expenditure during care-seeking for fever in South East Tanzania. BMC Health Serv Res.

[38] O’Connell KA, Poyer S, Solomon T, Munroe E, Patouillard E, Njogu J (2013). Methods for implementing a medicine outlet survey: lessons from the anti-malarial market. Malar J.

