# Supplementary material for: The malaria testing and treatment landscape in mainland Tanzania, 2016
Source: Malar J. 2017 Apr 24;16:202. doi: 10.1186/s12936-017-1819-7 (PMC5437635; doi:10.1186/s12936-017-1819-7)
Supplement: Supplementary file 1 — Additional file 1. Detailed breakdown of the sample. [file 12936_2017_1819_MOESM1_ESM.docx]

**Additional File 1: Detailed breakdown of the sample**

|  |  |  | **Public health facility*** | **Private not-for-profit** | **Public total** | **Private for-profit** | **Pharmacy** | **ADDO** | **DLDB** | **General Retailer** | **Itinerant vendor** | **Private total** | **Total** |
| --- | --- | --- | --- | --- | --- | --- | --- | --- | --- | --- | --- | --- | --- |
| **Number of outlets screened** | Urban | Booster | 89 | 31 | 120 | 134 | 48 | 814 | 0 | 0 | 0 | 996 | 1116 |
|  |  | Census | 45 | 18 | 63 | 54 | 12 | 381 | 81 | 2604 | 0 | 3132 | 3195 |
|  |  | Total | 134 | 49 | 183 | 188 | 60 | 1195 | 81 | 2604 | 0 | 4128 | 4311 |
|  | Rural | Booster | 151 | 11 | 162 | 17 | 0 | 231 | 0 | 0 | 0 | 248 | 410 |
|  |  | Census | 56 | 5 | 61 | 1 | 1 | 77 | 67 | 936 | 3 | 1085 | 1146 |
|  |  | Total | 207 | 16 | 223 | 18 | 1 | 308 | 67 | 936 | 3 | 1333 | 1556 |
|  | **Total** | | **341** | **65** | **406** | **206** | **61** | **1503** | **148** | **3540** | **3** | **5461** | **5867** |
| **Number of outlets eligible and interviewed** | Urban | Booster | 88 | 31 | 119 | 131 | 48 | 810 | 0 | 0 | 0 | 989 | 1108 |
|  |  | Census | 45 | 18 | 63 | 54 | 12 | 374 | 81 | 2 | 0 | 523 | 586 |
|  |  | Total | 133 | 49 | 182 | 185 | 60 | 1184 | 81 | 2 | 0 | 1512 | 1694 |
|  | Rural | Booster | 149 | 11 | 160 | 17 | 0 | 230 | 0 | 0 | 0 | 247 | 407 |
|  |  | Census | 54 | 5 | 59 | 1 | 1 | 76 | 65 | 10 | 3 | 156 | 215 |
|  |  | Total | 203 | 16 | 219 | 18 | 1 | 306 | 65 | 10 | 3 | 403 | 622 |
|  | **Total** | | **336** | **65** | **401** | **203** | **61** | **1490** | **146** | **12** | **3** | **1915** | **2316** |
| **Number of outlets eligible but not interviewed** | Urban | Booster | 0 | 0 | 0 | 0 | 0 | 2 | 0 | 0 | 0 | 2 | 2 |
|  |  | Census | 0 | 0 | 0 | 0 | 0 | 0 | 0 | 0 | 0 | 0 | 0 |
|  |  | Total | 0 | 0 | 0 | 0 | 0 | 2 | 0 | 0 | 0 | 2 | 2 |
|  | Rural | Booster | 0 | 0 | 0 | 0 | 0 | 0 | 0 | 0 | 0 | 0 | 0 |
|  |  | Census | 0 | 0 | 0 | 0 | 0 | 0 | 0 | 0 | 0 | 0 | 0 |
|  |  | Total | 0 | 0 | 0 | 0 | 0 | 0 | 0 | 0 | 0 | 0 | 0 |
|  | **Total** | | **0** | **0** | **0** | **0** | **0** | **2** | **0** | **0** | **0** | **2** | **2** |
| **Number of interviewed outlets with at least one anti-malarial in stock on the day of the survey** | Urban | Booster | 87 | 31 | 118 | 70 | 47 | 802 | 0 | 0 | 0 | 919 | 1037 |
|  |  | Census | 44 | 18 | 62 | 37 | 12 | 365 | 79 | 2 | 0 | 495 | 557 |
|  |  | Total | 131 | 49 | 180 | 107 | 59 | 1167 | 79 | 2 | 0 | 1414 | 1594 |
|  | Rural | Booster | 145 | 10 | 155 | 10 | 0 | 225 | 0 | 0 | 0 | 235 | 390 |
|  |  | Census | 54 | 5 | 59 | 1 | 1 | 75 | 63 | 8 | 2 | 150 | 209 |
|  |  | Total | 199 | 15 | 214 | 11 | 1 | 300 | 63 | 8 | 2 | 385 | 599 |
|  | **Total** | | **330** | **64** | **394** | **118** | **60** | **1467** | **142** | **10** | **2** | **1799** | **2193** |
| **Number of interviewed outlets with at least one anti-malarial in stock on the day of the survey or in the previous 3 months** | Urban | Booster | 88 | 31 | 119 | 71 | 48 | 810 | 0 | 0 | 0 | 929 | 1048 |
|  |  | Census | 45 | 18 | 63 | 37 | 12 | 374 | 81 | 2 | 0 | 506 | 569 |
|  |  | Total | 133 | 49 | 182 | 108 | 60 | 1184 | 81 | 2 | 0 | 1435 | 1617 |
|  | Rural | Booster | 148 | 11 | 159 | 11 | 0 | 230 | 0 | 0 | 0 | 241 | 400 |
|  |  | Census | 54 | 5 | 59 | 1 | 1 | 76 | 65 | 10 | 3 | 156 | 215 |
|  |  | Total | 202 | 16 | 218 | 12 | 1 | 306 | 65 | 10 | 3 | 397 | 615 |
|  | **Total** | | **335** | **65** | **400** | **120** | **61** | **1490** | **146** | **12** | **3** | **1832** | **2232** |
| **Number of interviewed outlets that provide malaria blood testing, but do not stock anti-malarial medicines** | Urban | Booster | 0 | 0 | 0 | 60 | 0 | 0 | 0 | 0 | 0 | 60 | 60 |
|  |  | Census | 0 | 0 | 0 | 17 | 0 | 0 | 0 | 0 | 0 | 17 | 17 |
|  |  | Total | 0 | 0 | 0 | 77 | 0 | 0 | 0 | 0 | 0 | 77 | 77 |
|  | Rural | Booster | 1 | 0 | 1 | 6 | 0 | 0 | 0 | 0 | 0 | 6 | 7 |
|  |  | Census | 0 | 0 | 0 | 0 | 0 | 0 | 0 | 0 | 0 | 0 | 0 |
|  |  | Total | 1 | 0 | 1 | 6 | 0 | 0 | 0 | 0 | 0 | 6 | 7 |
|  | **Total** | | **1** | **0** | **1** | **83** | **0** | **0** | **0** | **0** | **0** | **83** | **84** |
| **Number of interviewed outlets that reported distributing anti-malarials in the week prior to the survey** | Urban | Booster | 78 | 24 | 102 | 66 | 45 | 754 | 0 | 0 | 0 | 865 | 967 |
|  |  | Census | 41 | 18 | 59 | 31 | 12 | 339 | 72 | 2 | 0 | 456 | 515 |
|  |  | Total | 119 | 42 | 161 | 97 | 57 | 1093 | 72 | 2 | 0 | 1321 | 1482 |
|  | Rural | Booster | 133 | 8 | 141 | 9 | 0 | 209 | 0 | 0 | 0 | 218 | 359 |
|  |  | Census | 47 | 4 | 51 | 1 | 1 | 71 | 59 | 4 | 2 | 138 | 189 |
|  |  | Total | 180 | 12 | 192 | 10 | 1 | 280 | 59 | 4 | 2 | 356 | 548 |
|  | **Total** | | **299** | **54** | **353** | **107** | **58** | **1373** | **131** | **6** | **2** | **1677** | **2030** |
| **Number of interviewed outlets that reported providing/distributing a malaria diagnostic test in the week prior to the survey** | Urban | Booster | 70 | 28 | 98 | 117 | 0 | 36 | 0 | 0 | 0 | 153 | 251 |
|  |  | Census | 37 | 15 | 52 | 47 | 4 | 17 | 3 | 0 | 0 | 71 | 123 |
|  |  | Total | 107 | 43 | 150 | 164 | 4 | 53 | 3 | 0 | 0 | 224 | 374 |
|  | Rural | Booster | 98 | 7 | 105 | 14 | 0 | 22 | 0 | 0 | 0 | 36 | 141 |
|  |  | Census | 42 | 3 | 45 | 1 | 1 | 10 | 5 | 0 | 1 | 18 | 63 |
|  |  | Total | 140 | 10 | 150 | 15 | 1 | 32 | 5 | 0 | 1 | 54 | 204 |
|  | **Total** | | **247** | **53** | **300** | **179** | **5** | **85** | **8** | **0** | **1** | **278** | **578** |

* The breakdown of public health facilities that were interviewed in the study is as follows: National University hospitals, N=2; Regional Department hospitals, N=6; Health Zone Hospitals, N=12; Commune Health Centres, N= 25; Arrondissement Health Centres, N=188; Dispensaries, N=29, and Village Health Units, N=30, Total: N=310.
