# Supplementary material for: The malaria testing and treatment landscape in mainland Tanzania, 2016
Source: Malar J. 2017 Apr 24;16:202. doi: 10.1186/s12936-017-1819-7 (PMC5437635; doi:10.1186/s12936-017-1819-7)
Supplement: Supplementary file 2 — Additional file 2. QA AL availability in the public sector, by pack size. [file 12936_2017_1819_MOESM2_ESM.docx]

**Additional File 2: QA AL availability among all screened outlets in the public sector, by pack size**

|  | **Public**  **health facility**  **% (CI)** | **Private**  **not-for-profit**  **% (CI)** | **Public**  **Sector Total**  **% (CI)** |
| --- | --- | --- | --- |
|  | **N=312** | **N=48** | **N=360** |
| **QA AL 6 pack** | 62.2 | 23.2 | 57.9 |
|  | (51.0, 72.2) | (12.6, 38.9) | (47.6, 67.6) |
| **QA AL 12 pack** | 52.5 | 23.8 | 49.4 |
|  | (41.2, 63.5) | (14.6, 36.4) | (39.2, 59.6) |
| **QA AL 18 pack** | 55.8 | 22.4 | 52.1 |
|  | (44.0, 66.9) | (11.1, 40.0) | (40.8, 63.3) |
| **QA AL 24 pack** | 66.4 | 39.5 | 63.5 |
|  | (56.9, 74.7) | (24.1, 57.4) | (54.2, 71.9) |
