# Supplementary material for: The malaria testing and treatment landscape in mainland Tanzania, 2016
Source: Malar J. 2017 Apr 24;16:202. doi: 10.1186/s12936-017-1819-7 (PMC5437635; doi:10.1186/s12936-017-1819-7)
Supplement: Supplementary file 3 — Additional file 3. Availability of anti-malarials among all private sector screened outlets. [file 12936_2017_1819_MOESM3_ESM.docx]

**Additional File 3: Availability of anti-malarials among all private sector screened outlets**
