# Supplementary material for: The malaria testing and treatment landscape in mainland Tanzania, 2016
Source: Malar J. 2017 Apr 24;16:202. doi: 10.1186/s12936-017-1819-7 (PMC5437635; doi:10.1186/s12936-017-1819-7)
Supplement: Supplementary file 4 — Additional file 4. QA AL Availability in the private sector, by pack size. [file 12936_2017_1819_MOESM4_ESM.docx]

**Additional File 4: QA AL Availability among the anti-malarial stocking private sector, by pack size**

|  | **Private for-profit**  **Facility**  **% (CI)** | **Pharmacy**  **% (CI)** | **ADDO**  **% (CI)** | **DLDB**  **% (CI)** | **Private**  **Sector**  **Total**  **% (CI)** |
| --- | --- | --- | --- | --- | --- |
|  | **N=118** | **N=60** | **N=1468** | **N=142** | **N=1800** |
| **QA AL 6 pack** | 23.2 | 56.9 | 22.1 | 11.4 | 20.4 |
|  | (15.8, 32.7) | (49.9, 63.6) | (15.6, 30.3) | (4.7, 25.2) | (13.8, 29.2) |
| **QA AL 12 pack** | 18.1 | 41.3 | 20.8 | 15.6 | 19.3 |
|  | (9.3, 32.4) | (31.0, 52.4) | (15.6, 27.0) | (9.0, 25.8) | (13.8, 26.1) |
| **QA AL 18 pack** | 6.8 | 21.6 | 18.2 | 15.9 | 16.5 |
|  | (3.6, 12.5) | (16.1, 28.3) | (13.0, 24.9) | (8.2, 28.6) | (12.4, 21.7) |
| **QA AL 24 pack** | 64.3 | 75.3 | 53.7 | 44.8 | 52.7 |
|  | (54.2, 73.2) | (66.0, 82.7) | (46.9, 60.3) | (32.4, 57.9) | (46.2, 59.0) |
