# Supplementary material for: The malaria testing and treatment landscape in mainland Tanzania, 2016
Source: Malar J. 2017 Apr 24;16:202. doi: 10.1186/s12936-017-1819-7 (PMC5437635; doi:10.1186/s12936-017-1819-7)
Supplement: Supplementary file 5 — Additional file 5. Price for all private sector outlets. [file 12936_2017_1819_MOESM5_ESM.docx]

**Additional File 5: Median price for private sector outlets**

|  | **Private**  **for-Profit**  **Facility** | **Pharmacy** | **ADDO** | **DLDB** | **All**  **Private**  **Sector** |
| --- | --- | --- | --- | --- | --- |
|  | **(USD)**  **(IQR) ^(N)^** | **(USD)**  **(IQR) ^(N)^** | **(USD)**  **(IQR) ^(N)^** | **(USD)**  **(IQR) ^(N)^** | **(USD)**  **(IQR) ^(N)^** |
| **Non-**  **QAACT** | $5.58 | $5.58 | $4.65 | $1.67 | $4.65 |
|  | [4.19-7.50] ^(135)^ | [4.65-8.37] ^(205)^ | [1.40-6.25] ^(989)^ | [1.24-4.65] ^(51)^ | [1.55-6.25] ^(1,381)^ |
| **SP** | $1.40 | $1.40 | $1.05 | $1.05 | $1.05 |
|  | [0.93-1.40] ^(185)^ | [0.93-1.40] ^(193)^ | [0.93-1.40] ^(3,609)^ | [0.93-1.40] ^(241)^ | [0.93-1.40] ^(4,239)^ |
| **QAACT** | $1.86 | $1.86 | $1.40 | $1.40 | $1.40 |
|  | [1.40-2.79] ^(134)^ | [1.40-2.79] ^(130)^ | [1.24-1.86] ^(1,840)^ | [1.16-1.86] ^(140)^ | [1.24-1.86] ^(2,251)^ |

IQR: Interquartile range
