# Supplementary material for: The malaria testing and treatment landscape in mainland Tanzania, 2016
Source: Malar J. 2017 Apr 24;16:202. doi: 10.1186/s12936-017-1819-7 (PMC5437635; doi:10.1186/s12936-017-1819-7)
Supplement: Supplementary file 6 — Additional file 6. Antimalarial market share, within outlet type. [file 12936_2017_1819_MOESM6_ESM.docx]

**Additional File 6: Antimalarial market share, within outlet type**
