# Supplementary figures and images for: The malaria testing and treatment landscape in mainland Tanzania, 2016
Source: Malar J. 2017 Apr 24;16:202. doi: 10.1186/s12936-017-1819-7 (PMC5437635; doi:10.1186/s12936-017-1819-7)

**Additional File 7: SP product photographs from fieldwork**


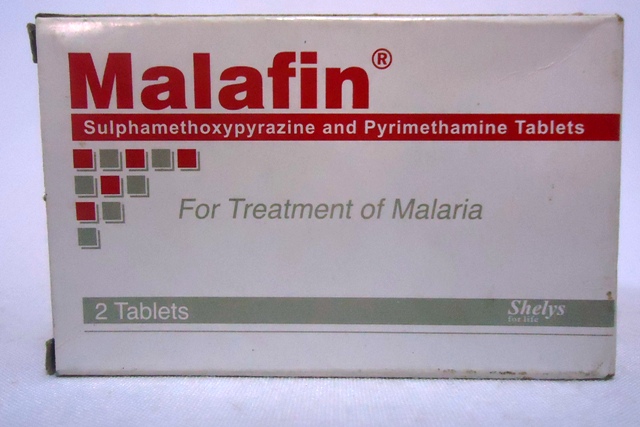


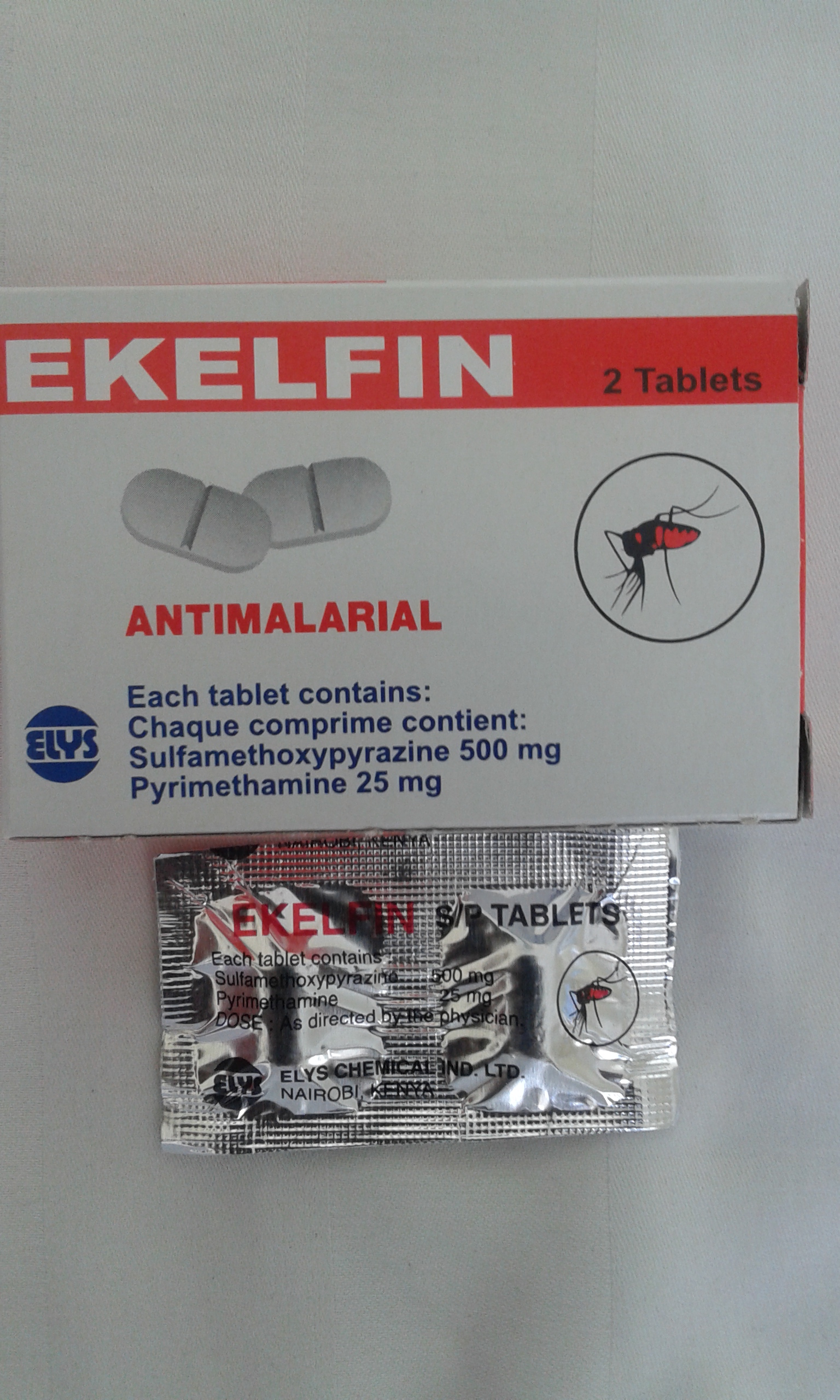


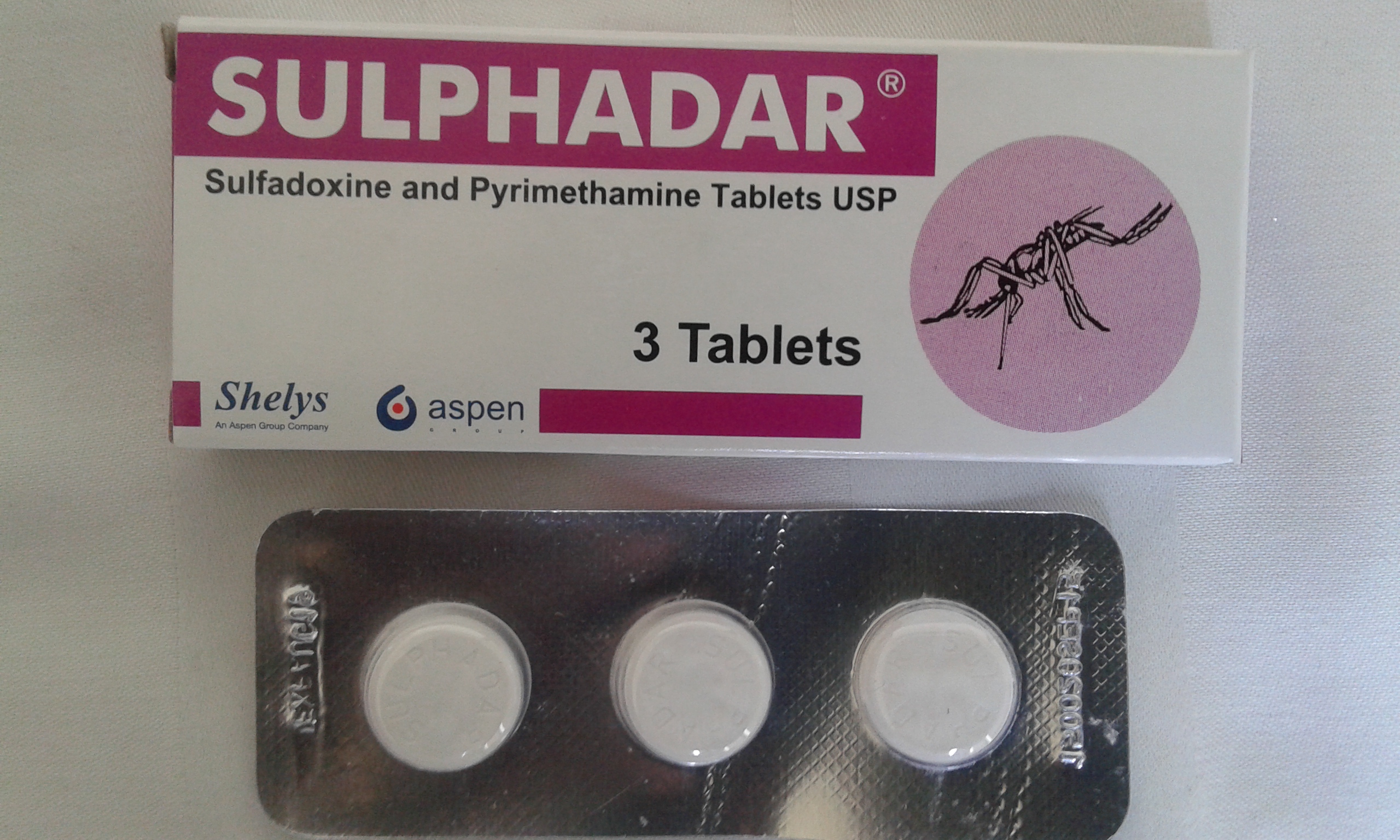

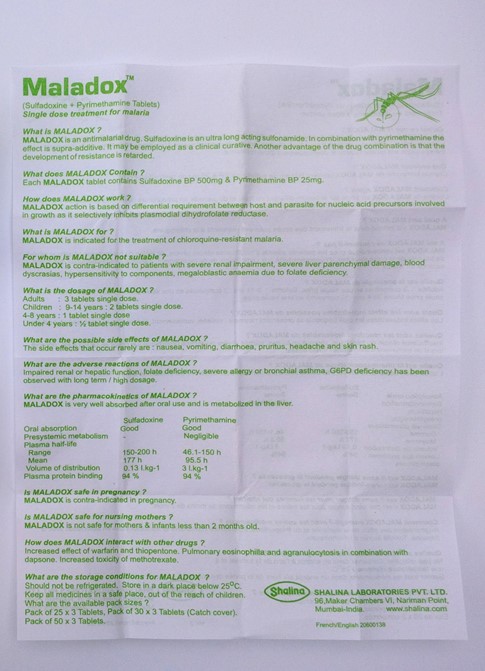

Supplement: Supplementary file 7 — Additional file 7. SP product photographs from fieldwork. [file 12936_2017_1819_MOESM7_ESM.docx]
